# A comparison of the effectiveness of QuEChERS, FaPEx and a modified QuEChERS method on the determination of organochlorine pesticides in ginseng

**DOI:** 10.1371/journal.pone.0246108

**Published:** 2021-01-29

**Authors:** Pennante Bruce-Vanderpuije, David Megson, Song-Hee Ryu, Geun-Hyoung Choi, Sang-Won Park, Byung-Seok Kim, Jin Hyo Kim, Hyo-Sub Lee

**Affiliations:** 1 Chemical Safety Division, National Institute of Agricultural Sciences, Rural Development Administration, Wanju-gun, South Korea; 2 CSIR Water Research Institute, Achimota, Accra, Ghana; 3 Ecology and Environment Research Centre, Manchester Metropolitan University, Manchester, United Kingdom; 4 Department of Agricultural Chemistry, Gyeonsang National University, Jinju, South Korea; University of Pisa, ITALY

## Abstract

This study provides a review of methods used in the determination of organochlorine pesticides (OCPs) in ginseng and compares the effectiveness of three extraction methods (Quick, Easy, Cheap, Effective, Rugged, and Safe (QuEChERS), a modified QuEChERS and a Fast Pesticide Extraction (FaPEx)) in the analyses of 20 OCPs in ginseng root samples. For each method, sample mass, solvent volume and sorbent mass were varied to identify the optimum combination to effectively isolate analytes of interest from the complex sample matrix. Extracts were analyzed using the gas chromatography-μ-electron capture detector (GC-μ-ECD), and confirmatory analyses performed by gas chromatography-tandem-mass spectrometry (GC-MS/MS). Eighteen out of 20 OCPs spiked onto in-house prepared ginseng samples produced acceptable recoveries (51–156%) when extracted using QuEChERS and FaPEx. All 20 analytes, including dichlorodiphenyldichloroethane (p, p’- DDD) and dichlorodiphenyltrichloroethane (o, p’-DDT), produced acceptable recoveries (51–129%) with the use of a modified QuEChERS method. The applicability of the modified QuEChERS method was demonstrated through the analysis of ginseng samples grown in endosulfan-treated soil. The samples were analyzed by both GC-μ-ECD and GC-MS/MS with no significant difference identified in the results of each analytical method. This study highlights the applicability of the modified QuEChERS method, in combination with GC- μ-ECD, to determine organochlorine pesticides in ginseng. This may be especially useful for laboratories in developing countries and less advanced institutions without access to MS/MS instrumentation.

## 1.0 Introduction

Pesticides control pests and increase crop yields, but their use has resulted in human health and environmental problems [1–3]. Pesticide exposure arises from direct routes such as occupational, household and agricultural usage, and indirectly via dietary intake [4, 5].

Globally, an estimated pesticide usage of approximately 6 billion pounds was observed in 2012 [6]. In most developing and emerging countries within Asia and the Americas, pesticide usage has been steadily increasing within the agricultural sector [7]. From 2010 to 2016, China (~14 kg/ha), Republic of Korea (~11 kg/ha) and Trinidad and Tobago (26 kg/ha) consumed the most average pesticide per area of cropland, in comparison to the United States of America (~3 kg/ha) [8]. Of the major classes of pesticides (insecticides, herbicides and fungicides), some organochlorines are included in the Stockholm Convention list of banned persistent organic pollutants (POPs). These chemicals are endocrine disruptors that negatively affect human reproduction and development [9–12]. Thus, pesticides with mutagenic, carcinogenic, reproductive toxicants and/or endocrine-disrupting potential are restricted, monitored, and/or regulated by governing regulatory systems [3].

In South Korea, past trends in pesticide management/regulation included the replacement of organochlorine pesticides- OCPs (1956–1980) with organophosphates, dithiocarbamates and chloroacetanilides [7]. In addition to the Stockholm Convention (ratified in Korea in 2001), the Pesticide Management Act, Pesticide Control Act, AgroChemicals Control Act, Toxic Chemicals Control Act and the POPs Control Act are utilized to set Korean national strategies for the control and management of OCPs [13]. Of international and national significance are the World Health Organization Maximum Residue Limit (MRL) and the Korean Positive List System (PLS) set at the lowest limit of analytical determination of 0.01 mg/kg, in an attempt to safeguard ingestion of pesticide residual contaminants from agricultural food crops [3, 13]. The toxicity of pesticides is known to affect organism within the ecosystem where it is applied; the degree of toxicity depends on the route of entry, dose and their properties [1]. Due to the toxic and carcinogenic nature of pesticides, their detection at trace levels in water, soil and other biological samples is of great importance [2]. Pesticides mainly contaminate water bodies through leaching from the soil [3], soil erosion, runoff from agricultural lands, amongst others [4]. DDD and DDT, two organochlorine pesticides known for their toxicity and persistence in the environment are of great concern to environmentalist as they have a severe effect on both target and non-target organisms [1]. It is remarkably noted that biotransformation leads to the formation of hazardous by-products [5]. In some extreme cases, the toxicity of pesticides has been correlated with incidences of nausea, abdominal pain and methemoglobinemia which most often is as a result of the disturbance to the reproductive, immune and the neurological systems of the human body [6].

Agricultural food crops, specifically root tubers, are cultivated in direct contact with the soil. In South Korea, ginseng is a major root tuber cultivated in eight of the nine Korean provinces; Korea is one of the world’s largest ginseng distributor [14]. Ginseng, a herbal medicinal root tuber, contains pharmacologically active ingredients including ginsenosides for the treatment of health-related diseases [15, 16]. Ginseng undergoes a long cultivation period (3–6 years). This increases the potential for uptake of toxic contaminants, such as OCPs, from soil contaminated with current and historic pesticide use. This increased risk necessitates the development of effective analytical methods for the quantitation of pesticides in agricultural food crops. A major challenge in the analyses of pesticide residues in ginseng root is the high complexity of the interfering matrix- consisting of saponins, polysaccharides, fatty acids and flavonoids. These are co-extracted alongside the low concentrations of pesticides, making accurate determination of OCPs difficult. To combat this, existing studies have combined extraction techniques with sensitive and/or selective advanced analytical methods (Table 1). Interfering ginseng matrix are often reduced with the use of isotopically labelled internal standards, matrix-matched calibration, clean-up steps, amongst other techniques (Table 1). However, disadvantages observed from the use of some of these techniques include false positive/negative (high/low) recoveries of some OCPs.

**Table 1:** A compilation of previous studies optimized for sample preparation analyses of pesticides in ginseng- extraction, clean-up and quantitation.

Class of organic pollutants	Sample preparation	Extraction technique	Clean-up technique	Instrumentation	Detected Pesticide concentration	Recoveries	Quantification	Endosulfan I, II, endosulfan sulfates, DDT (and metabolites quantified)	Reference
10 Organochlorine pesticides (OCPs)	Ginseng was ground using a mixer grinder	Supercritical Fluid Extraction (SFE) using CO_2_.	Solid-phase extraction (SPE) utilizing either 2g of: Silica gel, Neutral alumina or C_18_, **5 mL H**_**2**_**SO**_**4**_	^63^Ni GC-ECD Confirmation by GC-MS	Not specified LOD/LOQ: Not specified	**Recoveries from different solvent combinations:** ^**a**^ α-BHC: 69–190% β-BHC: 9–170% γ-BHC: 7–210% PCNB: 76–316%	Concentration of SFE was related to that of soxhlet extraction expressed as a %.	DDT and their metabolites were not detected with the use of SFE or soxhlet extraction	[31]
22 OCPs	Dried, pulverized sieved	QuEChERS- salting out	1 g Florisil SPE, Elute 10 mL hexane-acetone (95/5)	^63^Ni GC-ECD	Non-Detect (ND) to 1.51 mg/kg. LOD: 0.001–0.01 mg/ kg	80–105%	External calibration	o,p’-DDE, p,p’-DDE, o,p’-DDD, o,p’-DDT, p,p’-DDT, p,p’-DDD	[30]
6 OCPs	Fresh, dried and steamed ginseng	20 g ginseng, ACN, NaCl, exchange for hexane	**3 mL H**_**2**_**SO**_**4**_, vortex/centrifuge @ 2000 rpm, 500 mg Florisil SPE, elute 1.5 mL hexane, 10 mL ether/hexane (6/94)	^63^Ni GC-ECD	Not specified Blank ginseng samples were fortified and recoveries tested.	87.9–99.6%	External calibration	o,p’-DDE, p,p’-DDE, o,p’-DDD, o,p’-DDT, p,p’-DDT, p,p’-DDD; no endosulfans	[21]
168 pesticides including 13 OCPs	Dried ginseng powder	2 g ginseng + 10 mL H_2_O + ACN or acetone/cyclohexane/ethylacetate (2:1:1) + NaCl	C_8_ dispersive clean-up, SPE (Primary Secondary Amine (PSA-500 mg), Graphitized Carbon Black (GCB- 250 mg), Elution with acetone/toluene (3:1, 12 mL)	GC-MS GC-MS/MS	Fortified blank ginseng samples GC-MS: 0.005–0.333 mg/kg GC-MS/MS: 0.005–5 mg/kg	83–98%	Internal standard, Matrix-matched calibration	o,p’-DDE, p,p’-DDE, o,p’-DDD, o,p’-DDT, p,p’-DDT, p,p’-DDD	[22]
18 fungicides/ insecticide	Fresh ginseng	20 g ginseng + 100 mL ACN, NaCl,	Florisil SPE	^63^Ni GC-ECD, GC-NPD	LOD: 0.001–0.05 mg/ kg LOQ: 0.003–0.2 mg/kg	72.3–117.2%	Matrix-matched calibration (external calibration)	N/A	[23]
170 pesticides	Powdered ginseng roots	QuEChERS, Acetone, CAN	SPE- GCB, Aminopropyl silica, C18	GC x GC-TOF/MS, HR-TOF/MS GC-MS/MS, UPLC/Orbitrap-HR-MS, UPLC/MS/MS	LOD/LOQ not specified	> 50%	Matrix-matched, internal standard	N/A	[24]
32 pesticides	Fresh and dried ginseng	50 g ground ginseng + 100 mL ACN + NaCl, solvent exchanged for acetone/hexane	Florisil SPE	GC-MS/MS	LOD: 0.04–3.9 μg/kg LOQ: 0.15–13 μg/kg	55.2–108.3%	External calibration	p,p-DDE, p,p-DDT, p,p-DDD, o,p-DDT	[25]
42 pesticides	Ginseng tea	4 g + (H_2_O/ acetone, hexane: 2/4/4 mL) + 0.5 g MgSO_4_ + 2 g Na_2_SO_4_	dSPE: PSA, GCB, C_18_.	GC-MS	LOD: 0.15–4.00 μg/kg	65.5–109.5%	Matrix-matched calibration	p,p’-DDE, o,p’-DDT, p,p’-DDD, p,p’-DDT	[27]
103 organophosphorous pesticides	Dried ginseng root	2 g ginseng + 10 mL H_2_O + 15 mL acetonitrile + salting out	dSPE: C_18_ + MgSO_4_ + PSA + GCB	GC-FPD, GC-MS	LOD: N/A- 50 ng/g	70–120%	Matrix-matched calibration	N/A	[26]
135 pesticides: organophosphates, organochlorines, carbamates, pyrethroids	Dried ginseng root	2 g + (10 mL H_2_O/ 10 mL ACN) + 4 g MgSO_4_ + 1 g NaCN 2 g + (10 mL H_2_O/ 10 mL ACN) + 6 g MgSO_4_ + 1.5 g NaAc	SPE: 500 mg PSA/6 mL, elution (MeCN: toluene-20:1) dSPE: 50 mg PSA + 150 mg MgSO4	UHPLC-MS/MS	LOQ: 1–5 ng/g	90–110%	Matrix-matched calibration	N/A	[29]
116 pesticides Organophosphates	Ginseng root	2 g ginseng, 10 ml H_2_O, 10ml 0.1% formic acid in ACN, 4g MgSO4 + 1g NaCl	dSPE: 210 mg PSA + 70 mg GCB + 1.05 g MgSO4 + 200 mg C_18_	UPLC-ESI-MS/MS	LOQ: ≤ 10 ng/g	70–120%	Matrix-matched calibration	N/A	[28]

Cumulative summary of recoveries obtained from the use of varying solvent: n-hexane, 10% wt. CH_2_Cl_2_-n-hn-hexaneH_2_Cl_2_, and acetone

One of the most widely used methods for determining concentrations of organic pollutants in crops is the Quick, Easy, Cheap, Effective, Rugged and Safe (QuEChERS) method. This method has been used in combination with advanced analytical instrumentation such as gas or liquid chromatography coupled to a quadrupole mass spectrometer (GC-qMS) or a highly sensitive/selective mass spectrometer of high resolution, (e.g. GC-MS/MS, HPLC-MS/MS or GC-HRMS). In developing countries, there is not as much access to such instrumentation; thus, analyses by detectors such as Gas Chromatography-μ-Electron Capture Detector (GC-μ-ECD) is widely used. As the ECD is non-selective, it can be prone to interferences, which necessitates the use of thorough sample clean-up, such as a modified QuEChERS method for the determination of pesticides in complex samples such as root tubers. Traditional methods for multiclass extraction of pesticide residues in food include liquid-liquid extraction (LLE) and soxhlet extraction. These methods are reliable but can be slow and tedious; they are therefore gradually been replaced by the QuEChERS method for many applications [17–19]. Recently, a new method, Fast Pesticides Extraction (FaPEx), was developed on the principle of QuEChERS. It involves a microscale extraction with acetonitrile, and purification on a single-use pre-filled sorbent cartridge. This differs from QuEChERS which utilizes dispersive solid-phase extraction (dSPE) during the clean-up step [20]. With many different methods, it can often be a challenge to identify which is the most effective for a given application.

This study aimed to identify the most effective extraction method for 20 OCPs in ginseng and assess if this method could enable accurate quantitation by GC-μ-ECD. This was achieved through:

1. A review of publications on pesticide analyses in ginseng (analytical methods, instrumental detection and quantitation)
2. An assessment of the effectiveness of extraction/clean-up methods using the original QuEChERS method, a modified QuEChERS method, and a FaPEx method on the removal of interfering compounds, and the effects on analyte recoveries on ginseng control samples.
3. Demonstrating the successful application of the preferred extraction method using GC-μ-ECD and GC-MS/MS to determine OCP concentrations in ginseng grown in endosulfan-treated soil.

## 2.0 Materials and method

### 2.1 Materials, reagents and chemicals

Acetonitrile gradient grade for liquid chromatography LiChrosolv^®^ Reag. Ph Eur. was obtained from Merck, South Korea. SiliaPrep QuEChERS dispersive solid-phase extraction (d-SPE) clean-up kit for pigmented matrices (P/N 15662, 15 mL, pigmented fruits and cereals) and SiliaFast FaPEx pigmented (P/N FPX-PM-50, containing a dewating agent, C_18_, diamine and graphitized carbon black sorbents) were obtained from Silicycle KOREA LAB (Quebec, Canada). QuEChERS EN extraction kit (Bond Elut P/N 5982–0650, containing 4 g MgSO_4_, 1 g NaCl, 1 g Sodium Citrate Dihydrate, 0.5 g Sodium Hydrogencitrate Sesquihydrate) and QuEChERS d-SPE kit for fruit and vegetables (Bond Elut P/N 5982–5056, 15 mL, containing 150 mg of primary and secondary amine (PSA) and 900 mg of anhydrous MgSO_4_) were obtained from Agilent Technologies (Jeollabuk, South Korea). De-ionized water (18.2 MΩ) was produced by a PURELAB Option 2 system (Millipore, USA). Fifty millilitres (50 mL volume) polyethylene conical centrifuge tubes for initial extraction and 15 mL volume for d-SPE clean-up were obtained from Neurex Life Science (South Korea). VM-10 vortex mixer (Dathan Scientific), 514R Centrifuge (Hanil Science Industrial Combi), SPE extraction vacuum manifold (Supelco Visiprep), 1 and 10 mL Luer syringe (Henke Sass Wolf), and 13 mm 0.22 μm nylon syringe filter (RD Tech Research Development) were used in the sample extraction and clean-up. Gas Chromatographic column: DB5-MS (5% diphenyl 95% dimethyl polysiloxane, 30 m x 0.25 mm ID x 0.25 μm film thickness) was used in analyte separation.

### 2.2 Analytical standards

The OCP standards and their properties are listed in S1 Table in S1 File. The standards were obtained from Kemidas Chemicals as high purity analytical standards (> 95%) (South Korea). A stock standard solution (a mixture of 20 OCPs) of 10 mg L^-1^ was prepared in acetone. Working solutions for matrix-matched calibration and recovery test standards were prepared to a concentration range of 6.25 μg/L—2500 μg/L, and 1–10 mg/L, respectively. A native stock standard solution was diluted to produce a calibration curve set with a concentration range from 0.5 μg/L—500 μg/L. Pirimicarb-D_6_ PESTANAL^™^, analytical standard (purity ≥ 99.0%) was obtained from Sigma Aldrich (South Korea). Internal standard solution, pirimicarb-D_6_ of 2 mg/L was prepared in acetone and stored at 4°C before use. Ginseng control test materials were cultivated in pesticide-free soils in Rural Development Administration (RDA), Korea.

### 2.3 Sample preparation

In the absence of a National Institute of Standard Reference material for ginseng (fortified with OCPs), this study used in-house ginseng reference material prepared by spiking a control ginseng sample (5 g) with “low” (5 ng/g, 10 μL of 2.5 ng/μL spike), “medium” (50 ng/g, 10 μL of 25 ng/μL spike) and “high” (250 ng/g, 10 μL of 125 ng/μL spike) mass OCPs. It also included samples of ginseng grown in soil treated with commercial endosulfan thiolix dust (3%) (Low concentration treated soil: 0.1 mg/kg, high concentration treated soil: 1 mg/kg) produced under Rural Development Administration, Republic of Korea; project number: PJ013816. Before extraction, all ginseng samples were thoroughly washed under running water to remove soil, blended with dry ice and stored at -10°C.

#### 2.3.1 Organochlorine pesticides extraction from ginseng

**Fig 1:**
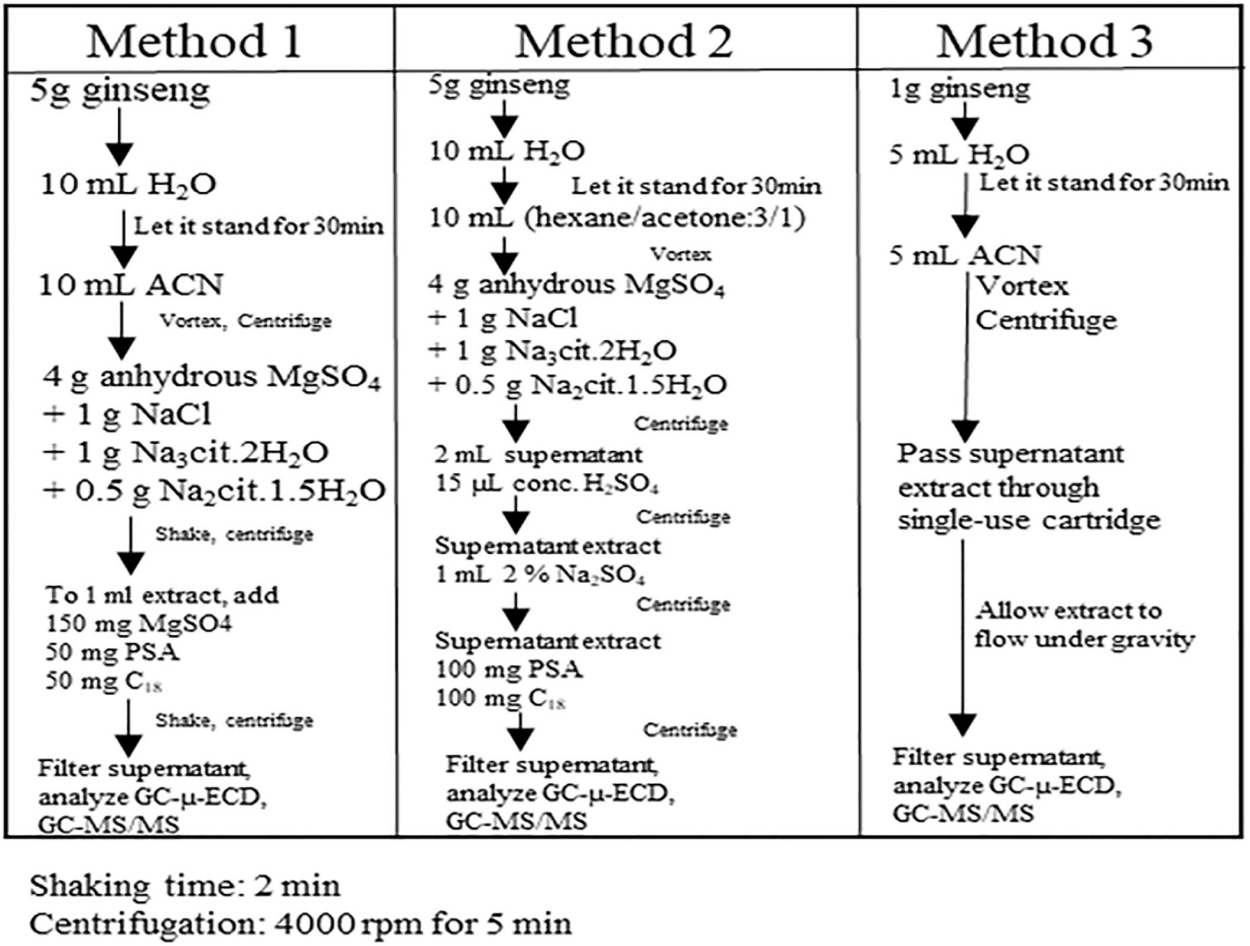
A schematic of the extraction and clean-up procedures for QuEChERS, modified QuEChERS and FaPEx methods used in ginseng analyses are explained. Extraction Method 1: QuEChERS, Method 2: modified QuEChERS, and Method 3: FaPEx Shaking time: 1–2 min; Centrifugation: 4000 rpm for 5 min. In FaPEx extraction, ginseng extract was allowed to flow under gravity during clean-up on the single-use cartridge.

A schematic of the extraction and clean-up procedures for QuEChERS, modified QuEChERS, and FaPEx are explained in Fig 1 below. A detailed explanation of the method development for the extraction and clean-up procedure to determine the extraction efficiency, matrix effects (ME) on analyte recovery, and enable quantitation by matrix-matched calibration, is presented in Supplementary information 1.0–1.3 in S1 File. The preparation of the matrix-matched calibration is detailed in Supplementary information 1.4 in S1 File. In brief, 5 g (QuEChERS and modified QuEChERS) and 1 g (FaPEx) ginseng control samples were spiked with 20 native OCP standards and Pirimicarb-D_6_; water was added, and samples were extracted using acetonitrile, and hexane/acetone. For clean-up, the QuEChERS citrate buffering salt was used for both the QuEChERS and modified QuEChERS methods; for FaPEx, a single-use pre-filled cartridge was used.

### 2.4 Instrumental analyses

Target pesticides were determined on a GC-μ-ECD, with confirmatory analyses performed by GC-EI-MS/MS. Method development was performed on a triple quadrupole mass spectrometer operating in MRM mode. A 1 μL sample extract was injected and chromatographically separated on a DB5-MS (30 m x 0.25 mm x 0.25 μm) for both GC-μ-ECD and GC-EI-MS/MS. Instrumental parameters and operating conditions are presented in Supplementary information 2.0 in S1 File.

#### 2.4.1 Quality control/quality assurance

The three extraction methods (QuEChERS, modified QuEChERS and FaPEx) were evaluated to determine their recoveries, linear ranges, and method detection limits (mDLs) on in-house prepared ginseng fortified with 20 OCP analytes. For linearity, matrix-matched calibration standards, prepared using blank extract samples, were fortified with calibration standards containing OCPs at a range of concentrations (6.25–2500 μg/L) as described in supplementary information 1.4 in S1 File. Analyte identification was based on the retention time for GC-μ-ECD and on the retention time and presence of two target ions in GC-MS/MS. A tolerance of ± 0.2 min was allowed for retention times; the allowed ratio of the MRM transition ions was set at a tolerance of ± 20%. The Limits of Detection (LOD) and Limit of Quantitation (LOQ) were calculated from the matrix-matched calibration curve, with 95% confidence, based on signal-to-noise ratio ≥ 3 (LOD) and ≥ 10 (LOQ), respectively for 20 OCP analytes.

The responses obtained, for the mean peak area of the 20 target analytes, relative to their corresponding concentrations were linear for the range of matrix-matched calibration standards analyzed. Calculated coefficient of determination for OCP analytes in GC-μ-ECD using the modified QuEChERS method was R^2^ ≥ 0.9995, with percent relative standard deviations (RSDs) ranging between 2.3 and 12.4% (these are in agreement with the acceptable EPA value of 15%).

## 3.0 Results and discussion

### 3.1 Review of existing methods utilized in ginseng analyses

A literature review was conducted using the following search terms; “ginseng”, “pesticides”, “organochlorine pesticides”, “OCPs”, “GC-MS”, “GC-MS/MS”, “GC-μ-ECD”, “sulphuric acid clean-up”, and “analytical instrumentation” on the web of science and google scholar. In total, 11 relevant papers were identified which were included for review; these are summarized in Table 1. A wide variety of sample preparation and clean-up methods have been used to determine trace contaminants of OCPs in agricultural products. Extraction techniques optimized in ginseng sample preparation have been developed based on a match between the target analyte solubility and solvent polarity, the ability of the solvent to limit extracting interfering co-extractants, and sorbent selectivity for the target analyte and their effect on analyte recoveries. From a comparison of extraction techniques in the analyses of pesticides in ginseng (Table 1), the method most commonly used was a modification of QuEChERS (73% of studies) [21–28], followed by QuEChERS (27% of studies) [22, 29, 30], and supercritical fluid with CO_2_ (9% of studies) [31]. Pesticides most commonly analyzed were OCPs (64% of studies), with organophosphates and carbamates also investigated. Sample types included fresh ground, dried ginseng roots, and ginseng tea. Ginseng sample masses extracted in previous studies ranged from 2 to 50 g. Some studies used sample pretreatment involving wetting the sample with water, and equilibrating for ≥ 30 min to improve recoveries [22, 26–29]. The nature and type of solvent, and extraction technique, are factors that influence the release of the analyte from the matrix and resultantly the analyte recovery. The choice of solvent used in the extraction of pesticides from ginseng has included a wide polarity range. The recoveries of ginseng organic extracts obtained from acetonitrile ranged from > 50 to 120%; recoveries obtained for a mixture of solvents- acetone/cyclohexane/ethylacetate, and hexane/acetone ranged from 83 to 98%, and 65 to 109%, respectively. Percentage recoveries obtained using supercritical fluid CO_2_ ranged from 7 to 316% [21–31]. Clean-up sorbents used in the studies reviewed included silica, florisil, C_18_, aminopropyl silicate, primary secondary amine (PSA), graphitized carbon black, and/or a mixture of some of these (Table 1). One of the studies reviewed utilized concentrated sulphuric acid (3–5 mL) in sample clean-up. In studies of Kim et al. (2008), ACN and NaCl were used in ginseng extraction, and sulphuric acid clean-up was used for the determination of 6 OCPs; recoveries ranged from ~88 to 100%. In a second study by Quan et al. (2004), DDTs and their metabolites could not be detected/quantified in ginseng with the use of supercritical fluid CO_2_, silica/C_18_/alumina and concentrated sulphuric acid clean-up [21, 31]. Analytical instruments used in the analyses of ginseng included GC-MS (21% of studies), GC-μ-ECD (21% of studies), GC-MS/MS (16% of studies), UPLC-MS/MS (11% of studies); other lesser-used instrumentations included GC-NPD, GC-FPD, UPLC-Orbitrap MS, UHPLC-MS/MS, GCxGC-TOF/MS, and the GC or HPLC-HRTOFMS (9% of studies). Pesticide quantitation involved the use of external calibration (25% of studies), internal standard (17% of studies) and matrix-matched calibration (58% of studies).

This review shows that a wide range of extraction and analytical methods have been used for the determination of OCPs in ginseng. The QuEChERS-type extractions appeared to generate better quality data than supercritical fluid extraction, especially when combined with the sulphuric acid cleanup. Therefore, the following three extraction methods (QuEChERS, modified QuEChERS and FaPEx) were investigated to establish the most effective method to determine OCPs in ginseng using GC-μ-ECD and GC-MS/MS.

### 3.2 Method development and optimization

For each method investigated (QuEChERS, modified QuEChERS and FaPEx), the following parameters were used in optimizing the sample preparation procedure- sample mass, solvent type and volume, extraction time, type and mass of sorbent, and the volume of concentrated sulphuric acid used for sample clean-up.

#### 3.2.1 Sample mass

Three sample masses (1 g, 5 g and 10 g) were tested during method development to observe the effect of the sample mass on matrix interferences and analyte recoveries. For QuEChERS and modified QuEChERS, a 5 g sample mass was identified as the optimum; however, with this mass, the majority of publications on OCPs and pesticides in Korean ginseng samples reported non-detect (nd) concentrations. By increasing the sample mass to 10 g, a reduction in response for some of the analytes of interest was identified in spiked samples. To improve extraction efficiency based on the correlation between the sample mass (1 g) and mass of extraction sorbent (~2.50 g) in the single-use extraction cartridge for the FaPEx method, a 1 g sample mass was considered optimum to avoid sample breakthrough in the cartridge. Therefore, for further method development work, 5 g sample mass was used for QuEChERS and modified QuEChERS, and a 1 g sample used for FaPEx.

#### 3.2.2 Sample pre-treatment

Wetting the sample with water (10 mL), and equilibrating and/or shaking for ≥ 30 min has been identified to improve the recoveries of moderately polar analytes [32]. For 1 g and 5 g ginseng samples, trials involved- no addition of water, the addition of water (10 mL) with equilibration, and addition of water with no sample equilibration. Vortexing of samples for 0 min, 2 min, 30 min, 1 hr, 5 hrs, and 24 hrs was also investigated. Analyte recoveries were slightly higher (+5%) for ginseng samples that were wetted with water, in comparison to those with no water addition. Sample pretreatment involving extensive vortexing (≥ 30 mins) after water addition did not significantly impact analyte recoveries. Therefore, for further method development work, 10 mL of water was added to ginseng and vortexed for 2 min, for QuEChERS and modified QuEChERS methods.

#### 3.2.3 Choice of solvent and extraction method

The original QuEChERS method [17, 19] involves the partitioning of OCP analytes into acetonitrile- a universal, water-miscible solvent (10 mL), and drying out using salts- MgSO_4_, NaCl, and citrate hydrates (Fig 1). Similarly, for FaPEx, ginseng extraction involved the use of acetonitrile (5 mL extraction volume, 5 mL elution) [20]. For the modified QuEChERS method, initial trials with n-hexane, a non-polar solvent, provided acceptable recoveries (50–110%) for the majority of the OCPs; low recoveries were obtained for some polar pesticides such as endosulfan sulfate, p, p’-DDT and heptachlor (≤ 40%). Thus, acetone (a solvent suitable for both polar and non-polar pesticides) was mixed with hexane in a bid to improve extraction. This resulted in improved recoveries (~51–129%) for a combination of hexane: acetone (3:1 *v/v*); these results are in agreement with published reports (although the ratio of hexane: acetone varied) [21, 25, 27, 33]. Therefore, for further method development work, hexane: acetone (3:1 *v/v*) was used for the modified QuEChERS method.

#### 3.2.4 Sample extraction time—Vortexing and centrifugation

An optimum extraction time allows for sufficient interaction and transport of the bound analyte from the matrix into the extraction solvent. The following conditions were selected based on the review of the existing literature; vortex for 2 min, and centrifuge at 4000 rpm for 5 min. Although the effect of sonication has been shown to improve recoveries slightly, it also adds time to the sample preparation step, which was deemed unsuitable for the development of a rapid extraction method. Therefore, for further method development work, extracts from all three methods were vortexed for 2 min and centrifuged at 4000 rpm for 5 min.

#### 3.2.5 Clean-up method optimization

Ginseng matrix, known to contain saponins, polysaccharides and fatty acids, make it difficult to quantify pesticide residues as some pesticides are bound to the interfering matrix. Initial trials on the type of clean-up involved the use of dSPE and SPE. Irrespective of the sorbent used, SPE extracts were identified to be much cleaner than dSPE, although the disadvantage with SPE is that it takes longer due to column conditioning and elution (gravity or under vacuum). The following clean-up methods were investigated: graphitized carbon black (GCB), primary secondary amine (PSA), C_18_, silica/florisil, and sulphuric acid.

Graphitized carbon black is useful in the removal of pigments from extracts, although it can reduce recoveries of the planar analyte- hexachlorobenzene (potentially as a result of its high affinity towards GCB). GCB has been previously used in some studies determining OCPs in ginseng; however, trials with GCB in this current study produced recovery of ~45% for HCB. Hence, GCB was not used in further optimization.

Primary secondary amine is known to remove interfering fatty acids and has previously been combined with C_18_ (non-polar sorbent removes non-polar interferences) in dispersive solid-phase extraction (dSPE) [22, 26–29]. Various mass combinations of PSA and C_18_ (0, 50, 100, 150, 250 mg) were investigated. It was identified that a combination of PSA (100 mg) and C_18_ (100 mg) was optimum at effectively removing interferences from ginseng extract for both QuEChERS and modified QuEChERS methods. Trials with PSA and C_18_ masses greater than 100 mg had no additional effect on improving analyte recoveries.

Trials with silica and a combination of silica/florisil (100, 250 and 500 mg) proved ineffective at removing the matrix components in ginseng. The extraction recoveries observed at the medium (50 ng) and high spike concentrations (250 ng) for QuEChERS and FaPEx ranged between ~74–156% and 111–135%, respectively. At the low spike level (5 ng), all three methods were inefficient at producing acceptable recoveries (≥ 50%) due to the complexity of the matrix-bound effect on two target analytes- dichlorodiphenyldichloroethane (p,p’- DDD) and dichlorodiphenyltrichloroethane (o, p’- DDT).

Several studies have shown that a combination of dSPE or SPE, and sulphuric acid (dSPE/H_2_SO_4_, SPE/H_2_SO_4_), is useful for the removal of matrix components in complex samples for pesticides including organochlorine pesticides [21, 31, 34]. Although sulphuric acid is known to degrade OCPs such as aldrin, dieldrin, endrin, α-, β- endosulfan, and endosulfan sulfate [35], it precipitates interfering matrices, results in lesser interferences and gives acceptable recoveries (≥ 50%).

Dispersive SPE clean-up of QuEChERS and modified QuEChERS and SPE clean-up for FaPEx produced interferences for two target analytes (p, p’-DDD and o, p’- DDT). Therefore, the addition of sulphuric acid to precipitate matrix components in ginseng was considered necessary. Varying volumes of dilute sulphuric acid in combination with dSPE were tested to identify the optimum volume for matrix precipitation. The use of concentrated sulphuric acid (10–500 μL) was avoided as it was identified to degrade analytes; dieldrin, α-, β- endosulfan, and endosulfan sulfate. Differing volumes (0, 10, 15, 25, 50, 100, 250, 500, 1000 μL) of 50% sulphuric acid (in H_2_O) were added to 2 mL hexane extract to calculate the influence of sulphuric acid in precipitating the matrix and improving the efficiency of the recovery. Fifteen microlitres of 50% sulphuric acid was identified as optimum, as volumes above 15 μL deteriorated some OCPs (endrin, α-, β- endosulfan, and endosulfan sulfate), which resulted in recoveries below 50%. Therefore, a final clean up method of dSPE with 15 μL of 50% sulphuric acid was selected.

### 3.3 Matrix effect

To reduce interfering matrix and ME, several techniques have been employed including sample dilution (signal suppression), the selectivity of the chromatographic separation (eliminates issues of co-elution of the analyte of interest with the interfering matrix), the use of isotopically labelled internal standard, or the use of a matrix-matched calibration [36–40]. The presence of co-extractives, evidenced by differences in slopes of the calibration curve for the pure standard in the solvent, and the standard in the matrix extract (matrix-matched calibration curve) for each analyte, can be attributed to a systematic error caused by the matrix. Considering the influence of signal suppression (slope in matrix-matched calibration is lower than in pure standard = negative), signal enhancement (slope in matrix-matched calibration is higher than in pure standard = positive), and 100% ME (slope in matrix-matched calibration = slope in pure standard), the ME was evaluated by comparing the slope of the matrix-matched standard (1–100 μg L^-1^) with the corresponding slope observed in the pure standard calibration curve (1–100 μg L^-1^) in seven replicates. The ME was calculated as the average percent enhancement or suppression in the slopes using the equation:

$$\%\;\text{ME}=\frac{Slope\;of\;matrix-matched\;calibration\;curve}{Slope\;of\;standard\;in\;solvent}\times 100\%$$

The % ME ranged from 68 to 155%, although four analytes exhibited matrix enhancements (≥ 100%)—delta BHC, endrin, endosulfan sulfate, and p, p’- DDT; this indicates that at higher concentrations (≥ 25 μg L^-1^), the matrix effect is not quite significant. However, at lower concentrations (≤ 5 μg L^-1^), a pronounced effect of the matrix is experienced in the analysis of OCPs in ginseng, as two of the analytes (p, p’- DDD and o, p’-DDT) co-elute with the interfering matrix at retention times 29.59 and 29.83 min (Fig 3), respectively. This presents a challenge for low-level analyses of OCPs in ginseng (and other root tubers) for both the GC-μ-ECD and GC-MS/MS.

### 3.4 Comparison of QuEChERS, modified QuEChERS and FaPEx methods

#### 3.4.1 Quality assurance/quality control

**Fig 2:**
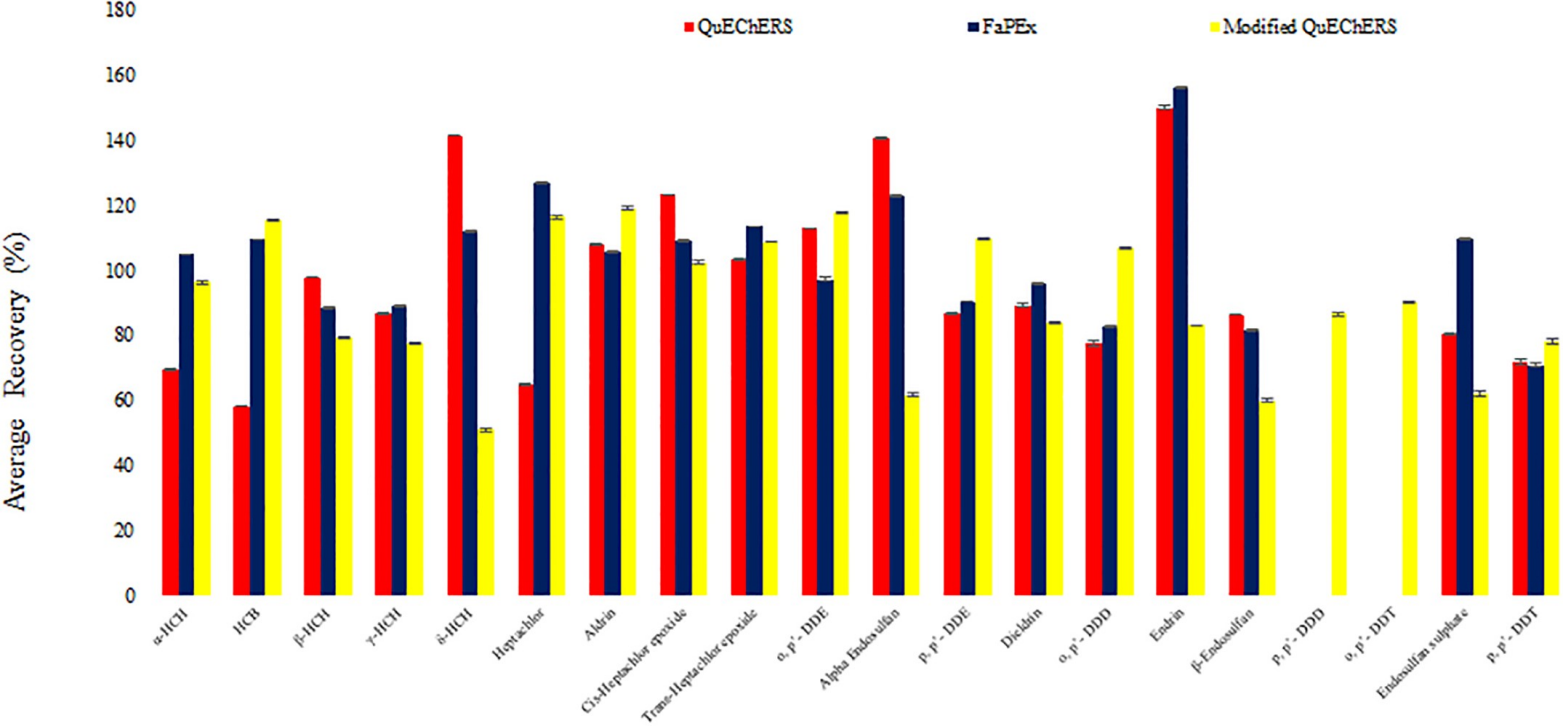
Average recoveries (n = 3) for blank ginseng spiked at 5 ng/g, and analyzed using three extraction/clean-up methods: QuEChERS, modified QuEChERS and FaPEx. ***- Matrix interference: The effect of the matrix is masked at higher concentrations (50 ng/g and 250 ng/g). At the lower concentration (5 ng/g), the intensity of the interfering matrix is pronounced, and analytes: p, p’-DDD, and o, p’-DDT cannot be accurately quantified with the use of QuEChERS and FaPEx, thus the need for acid clean-up. Analyte quantitation was with GC-μ-ECD.

The responses obtained, for the mean peak area of the 20 target analytes, relative to their corresponding concentrations were linear for the range of matrix-matched calibration standards analyzed. The calculated coefficient for determination of OCP analytes in GC-μ-ECD using the modified QuEChERS method was R^2^ ≥ 0.9995, with percent relative standard deviations (RSDs) ranging between 2.3 and 12.4% (these are in agreement with the acceptable EPA value of 15%). Recovery concentrations for blank ginseng were fortified at three levels 5, 50 and 250 ng/g. These were quantified from the matrix-matched calibration curve using the peak area of the analytes in the spiked blank samples. The recoveries of the analytes fortified at three levels ranged between 51–156% for QuEChERS and FaPEx methods, except for 2 analytes (recoveries could not be obtained for 5ng/g spike for p, p’- DDD and o, p’-DDT). Additionally, the calculated R^2^ was ≥ 0.9997, with RSDs ranging between 1.8% and 12.6%. Results of recovery and limits of detection/quantitation are tabulated in Table 2. Fig 2 shows the average recoveries (n = 3) for blank ginseng spiked at 5 ng/g, and analyzed using three extraction/clean-up methods: QuEChERS, modified QuEChERS and FaPEx. The results of the LOD and LOQ for the target analytes, obtained for the optimum method (modified QuEChERS), using the GC-μ-ECD, are presented in Table 2. The LOD ranged between 0.18 and 2 ng/g, and LOQ ranged between 0.55 and 6 ng/g.

**Table 2:** Optimized GC-MS/MS and GC-μ-ECD conditions for 20 organochlorine pesticides analyzed; % recoveries for QuEChERS, modified QuEChERS and FaPEX methods in ginseng, limit of detection and quantitation from GC-μ-ECD.

Analyte	GC-μ-ECD Retention time	GC-MS/MS Retention time	Precursor ions (m/z)	Product ions (m/z) [Collision Energy (eV)]	% Recovery QuEChERS method, n = 3, sample mass = 5 g			% Recovery modified QuEChERS method, n = 3, sample mass = 5 g			% Recovery FaPEx method, n = 3, sample mass = 1 g			GC-μ-ECD Modified QuEChERS	
					5 ng/g	50 ng/g	250 ng/g	5 ng/g	50ng/g	250 ng/g	5 ng/g	50 ng/g	250 ng	LOD (ng/g)	LOQ (n/ g)
α-HCH	13.65	8.1	181.0	145.0 [15], 109.0 [30]	69.3	119.5	143.1	96.1	98.3	123.7	104.7	128.4	120.1	0.36	1.09
HCB	13.91	9.5	283.9	248.8 [25], 213.9 [35]	58.2	108.5	130.7	115.5	124.7	124.1	109.4	139.2	127.4	0.25	0.76
β-HCH	14.47	9.8	181.0	145.0 [15], 109.0 [30]	97.8	109.4	128.0	79.3	97.6	95.7	88.4	134.6	120.7	0.35	1.05
γ-HCH	14.70	9.9	181.0	145.0 [15], 109.0 [30]	86.7	105.5	124.7	77.7	76.2	115.9	88.7	141.1	119.6	0.28	0.85
δ-HCH	15.53	10.4	181.0	145.0 [15], 109.0 [30]	141.4	123.9	156.0	50.9	72.6	63.9	112.0	150.2	128.7	0.31	0.92
Heptachlor	17.51	11.6	271.9	236.8 [25], 116.9 [40]	64.8	111.0	153.3	116.2	115.0	123.6	126.6	140.8	119.1	0.58	1.76
Aldrin	19.27	12.6	262.9	192.9 [40], 190.9 [15]	107.8	113.2	130.4	85.0	92.9	95.2	105.4	141.5	117.1	0.38	1.15
Cis-Heptachlor epoxide	21.72	13.7	183.0	154.9 [15], 118.9 [30]	123.1	106.2	138.9	102.4	103.9	111.6	109.0	149.6	121.1	0.50	1.52
Trans-Heptachlor epoxide	22.01	13.9	352.9	281.9 [20], 262.8 [25]	103.2	101.0	133.8	108.7	103.7	109.2	113.4	147.6	121.1	0.43	1.30
o, p’- DDE	23.70	14.6	246.0	211.1 [20], 176.1 [40]	112.8	104.4	128.0	117.6	114.7	111.8	97.2	140.5	114.9	0.85	2.56
Alpha Endosulfan	24.33	14.8	240.9	205.9 [15], 136.0 [40]	140.4	113.7	144.8	61.5	116.1	119.0	122.8	148.3	118.9	0.18	0.55
p, p’- DDE	26.24	15.6	246.0	176.1 [40], 175.1 [40]	86.5	103.9	136.8	109.4	118.1	99.0	90.3	145.5	115.7	0.92	2.79
Dieldrin	26.44	15.7	262.9	192.9 [40], 190.9 [40]	89.2	103.0	137.4	83.6	96.9	108.2	95.6	147.9	119.0	1.04	3.14
o, p’- DDD	26.97	15.9	235.0	199.1 [15], 165.1 [30]	77.5	89.6	105.8	106.6	81.9	95.9	82.6	143.5	113.8	0.48	1.45
Endrin	28.16	16.4	262.9	193.0 [35], 190.9 [35]	150.0	101.2	141.0	83.0	84.9	109.9	156.1	154.0	119.3	0.76	2.30
β-Endosulfan	28.90	16.7	240.9	205.9 [40], 136.0 [40]	86.3	91.2	143.6	59.9	102.4	70.9	81.4	150.2	121.6	0.85	2.58
p, p’- DDD	29.59	17	235.0	199.1 [15], 165.1 [30]	***	92.5	108.9	86.5	77.0	113.7	***	139.0	110.9	0.35	1.06
o, p’- DDT	29.83	17.1	235.0	199.1 [15], 165.1 [30]	***	74.2	76.5	90.2	90.2	126.3	***	137.5	134.6	0.82	2.48
Endosulfan sulphate	32.09	18.1	271.9	236.9 [20], 116.9 [40]	80.1	115.2	141.8	62	70.3	97.4	109.7	147.8	118.5	2.00	6.06
p, p’- DDT	32.37	18.2	235.0	199.1 [15], 165.1 [30]	71.6	114.4	118.0	78	79.6	128.8	70.7	150.3	128.1	2.00	6.06

***- Matrix interference: The effect of the matrix is masked at higher concentrations (50 ng/g and 250 ng/g). At lower concentrations (≤ 5 ng/g), the intensity of the interfering matrix is pronounced, and analytes: p, p’-DDD, and o, p’-DDT cannot be accurately identified/quantified.

The use of QuEChERS, FaPEx and modified QuEChERS methods generally provided efficient recoveries (~51–156%) at medium and high spike concentrations (50 ng/g and 250 ng/g). The need for efficient quantitation of DDTs and their metabolites, α-, β-endosulfan and endosulfan sulfate in ginseng, stem from concerns of the possible presence from past OCP usage (over four decades ago) in agricultural soil in South Korea. Thus, the optimum method, identified to remove interfering complex matrix to allow for accurate determination of p, p’- DDD and o, p’- DDT was the modified QuEChERS method in combination with sulphuric acid clean-up.

The optimized modified QuEChERS method required a sample mass of 5 g, 10 mL water for equilibration for ≥ 30 min, 10 mL hexane: acetone (3:1 *v/v*), QuEChERS pouch salt, centrifugation at 4000 rpm for 5 min, and 15 μL of 50% sulphuric acid (in H_2_O).

**Fig 3:**
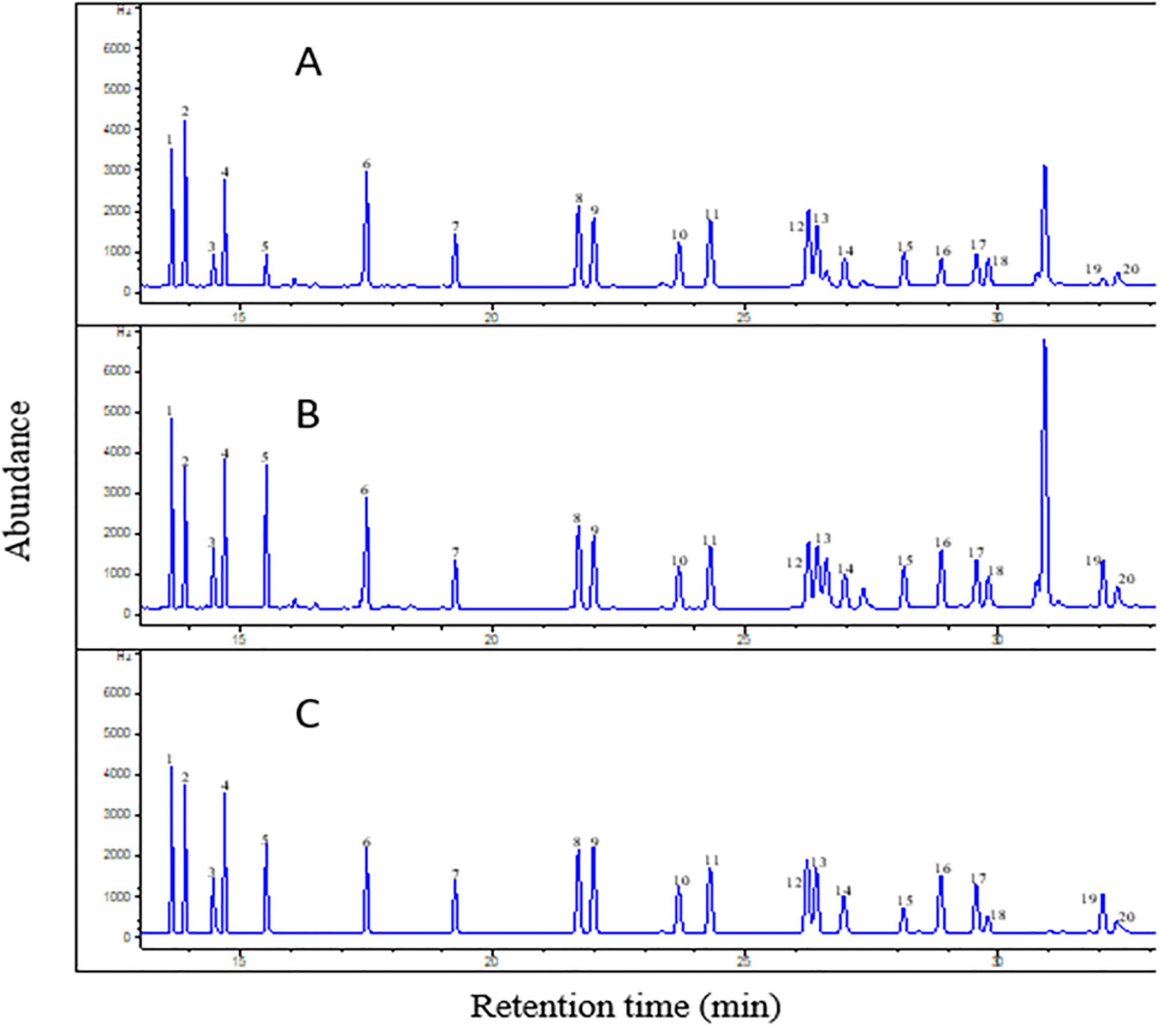
GC-μ-ECD chromatograms of- A: 50 μg/Lfortified ginseng blank extract containing 20 organochlorine pesticide (OCP) mix; B: 50 μg/L matrix-matched standard- ginseng blank was extracted, 10 μL of 1250 μg/L standard mix (20 OCP analytes) was added to 240 μL ginseng blank extract; C: 50 μg/L organochlorine pesticide standard mix (20 compounds), using the modified QuEChERS method. List of OCP analytes- 1: α-HCH, 2: HCB, 3: β-HCH, 4: γ-HCH, 5: δ-HCH, 6: Heptachlor, 7: Aldrin, 8: Cis-Heptachlor epoxide, 9: Trans-Heptachlor epoxide, 10: o, p’- DDE, 11: Alpha Endosulfan, 12: p, p’- DDE, 13: Dieldrin, 14: o, p’- DDD, 15: Endrin, 16: β-Endosulfan, 17: p, p’- DDD, 18: o, p’- DDT, 19: Endosulfan sulfate, 20: p, p’- DDT.

Extracts were washed in 2% Na_2_SO_4_ solution, centrifuged, and the supernatant filtered before dSPE/C_18_ clean-up for modified QuEChERS. Fig 3 shows the chromatograms obtained using the modified QuEChERS method comparing ginseng blank fortified at A: 50 ng/g spike, B: 50 ng matrix-matched standard, and C: 50 ng/g OCP standard mix.

### 3.5 Application of the optimized method on cultivated ginseng samples

The modified QuEChERS method was identified as the preferred method for determining OCPs in ginseng. Its applicability to determine OCP concentrations in ginseng samples was demonstrated through analyses of ginseng grown in soil treated with α-, β-endosulfan, and endosulfan sulfate. The effectiveness of the method was assessed by comparing the results against extracts prepared using the traditionally accepted method QuEChERS with analysis by GC-MS/MS. A total of 9 samples were prepared from ginseng grown in soil with no endosulfan (control, n = 3), “low” endosulfan-treated soil (0.1 mg/kg, n = 3), and “high” endosulfan-treated soil (1 mg/kg, n = 3) 2 years.

#### 3.5.1 Endosulfan (α-, β) and endosulfan sulfate concentrations in ginseng

The mean α-endosulfan concentrations for a low and high-concentration treated soil obtained using the traditional QuEChERS method and GC-MS/MS were 0.011 ± 0.002 mg/kg and 0.28 ± 0.003 mg/kg, respectively. When compared to the modified QuEChERS method with analysis by GC-μ-ECD, concentrations were 0.023 ± 0.001 mg/kg (low), and 0.230 ± 0.003 mg/kg (high). From these results, there was no statistically significant difference between the two methods (p-value = 0.007). Similar results were obtained for β-endosulfan and endosulfan sulfate; there was no statistically significant difference for the two methods compared at low and high concentration treated soil. The results are detailed in S1 Fig in S1 File. For the control samples (blank), OCPs were not present. These results indicate that it is possible to successfully determine OCPs in ginseng using GC-μ-ECD. By using the modified QuEChERS method along with a more extensive clean up involving sulphuric acid, it was possible to generate similar data to that produced by the traditional method of QuEChERS and GC-MS/MS. This finding may be especially useful for developing countries and institutions which do not have access to advanced analytical instrumentation.

## 4.0 Conclusions

The motivation for this study was to establish if an optimized method could allow for accurate determination of OCPs in a complex matrix (ginseng) using GC-μ-ECD. Eleven papers were reviewed to identify suitable candidate methods for analyses. Three extraction methods were identified for further investigation: QuEChERS, modified QuEChERS and FaPEx. As ginseng is a complex sample matrix, a variety of clean-up methods were also investigated- graphitized carbon black (GCB), primary secondary amine (PSA) and C_18_, silica/florisil, and sulphuric acid.

Ginseng root tubers (control samples) were fortified with 5, 50 and 250 ng/g (20 OCP native analytes) and used to investigate the effectiveness of each technique. From the results, we identified that QuEChERs and FaPEx methods were able to provide acceptable recoveries (> 50%) for 18 of the 20 OCPs tested. The modified QuEChERS method was capable of providing acceptable recovery rates for all 20 OCPs.

The effectiveness of the modified QuEChERS method was tested against the traditional QuEChERS method with GC-MS/MS analysis. A total of 9 ginseng samples grown in untreated, and endosulfan-treated soil (0.1 mg/kg and 1 mg/kg) were extracted and analyzed by each method. The results showed no statistically significant difference between samples prepared by QuEChERS with GC-MS/MS analysis and those prepared by modified QuEChERS and GC-μ-ECD analysis. This highlights the applicability of this modified QuEChERS method to determine OCPs in crops and may benefit those in less well-funded institutions and developing countries who do not have access to advanced analytical instrumentation.

## Supporting information

S1 File (DOCX)
