# Supplementary material for: A comparison of the effectiveness of QuEChERS, FaPEx and a modified QuEChERS method on the determination of organochlorine pesticides in ginseng
Source: PLoS One. 2021 Jan 29;16(1):e0246108. doi: 10.1371/journal.pone.0246108 (PMC7846022; doi:10.1371/journal.pone.0246108)
Supplement: S1 File — (DOCX) [file pone.0246108.s001.docx]

A comparison of the effectiveness of QuEChERS, FaPEx and a modified QuEChERS method on extraction of organochlorine pesticides in Korean ginseng.

Pennante Bruce-Vanderpuije,^1,2^ David Megson,^3^ Song-Hee Ryu,^1^ Geun-Hyoung Choi,^1^ Sang-Won Park,^1^ Byung-Seok Kim,^1^ Jin Hyo Kim,^4^ Hyo-Sub Lee.^1*^

SI 1.0 QuEChERS and FaPEx Method Development- Analytical procedure

Prior to sample extraction, method development was with control ginseng. For method detection limits, 5 g control ginseng was weighed into 24 x 50 mL centrifuge tubes for QuEChERS (n=3) and modified QuEChERS method (n=3); 1 g control ginseng was weighed into 12 x 15 mL centrifuge tubes for FaPEx (n=3). Control ginseng samples (a set of 7 samples was used as control for QuEChERS, modified QuEChERS and FaPEx methods) were fortified with a mixture of 20 native OCP standards at three spiking levels in 7 replicates: 5 ng, 50 ng, and 250 ng, to determine the extraction efficiency, matrix effects on recovery, and enable quantitation by matrix-matched calibration. For QuEChERS and modified QuEChERS- 10 mL, and FaPEx- 5 mL water were added, respectively; sample mix was vortexed and allowed to stand for 30 min at room temperature. Ten milliliter (10 mL) and 5 mL ACN were added for QuEChERS and FaPEx samples, 10 mL (hexane: acetone- 3:1 v/v) was added for modified QuEChERS samples. Samples were vortexed for 2 min to extract analytes. The QuEChERS EN extraction kit- citrate buffering salt pouch (5982-0650) was added to each tube for QuEChERS and modified QuEChERS, shaken vigorously to prevent agglomeration, and vortexed for 2 min. Samples were centrifuged at 4000 rpm for 5 min to separate the organic and aqueous layers.

SI 1.1 QuEChERS Ginseng sample clean-up

One milliliter (1 mL) ginseng organic layer extract (in ACN) was transferred into a 2-mL mini dispersive solid-phase extraction (dSPE: 5982-5021 Fruit and veg EN) clean-up tube, shaken for 2 min, and vortexed at 4000 rpm for 5 min. Extract was filtered and 1 mL was analyzed on the GC-µ-ECD and GC-MS/MS.

SI 1.2 Modified QuEChERS Ginseng sample clean-up

To 2 mL ginseng organic layer extract, 15 µL 50 % sulphuric acid was added to precipitate the saponin and polysaccharide matrix in ginseng, vortexed and centrifuged at 4000 rpm for 1 minute. The supernatant was washed in 2 % sodium sulphate solution (Na_2_SO_4_) and cleaned up by dSPE using 100 mg PSA and 100 mg C_18_; the extract was vortexed and centrifuged at 4000 rpm for 5 min. The cleaned-up extract was filtered and 1 mL was analyzed on the GC-µ-ECD and GC-MS/MS.

SI 1.3 FaPEx Ginseng sample clean-up

Ten milliliter (10 mL) of the extract was passed through a single-use prefilled solvent cartridge and allowed to flow under gravity; the extract was analyzed on the GC-µ-ECD and GC-MS/MS.

SI 1.4 Preparation of Matrix-matched calibration

The working solution, containing a mixture of 20 organochlorine pesticides, was diluted to concentration levels: 6.25, 12.5, 25, 62.5, 125, 250, 625, 1250, and 2500 µg L^-1^. To 240 µL ginseng control matrix extract in inset vials, obtained from SI 1.1, 1.2, 1.3, 10 µL of each standard was added. The resulting matrix-matched calibration for ginseng were 0.25, 0.5, 1, 2.5, 5, 10, 25, 50, and 100 µg L^-1^ for each method studied.

SI 2.0 Instrumentation

Target pesticides were separated on a DB-5MS (30 m x 0.25 mm x 0.25 µm- for GC-µ-ECD) and confirmation by GC-EI-MS/MS. For GC-µ-ECD, the injector and detector temperatures were set at 250 °C in splitless mode, and 300 °C, respectively. Nitrogen was used as the carrier gas and make-up gas at a constant flow rate of 6.0 mL min^-1^, with ≥ 99.999 % purity. The initial oven temperature programme was 80 °C (2 min hold); followed with an increase of 20 °C min^-1^ to 180 °C (2 min hold), then 5 °C min^-1^ to 205 °C (2 min hold); 1 °C min^-1^ to 207 °C (3 min hold), 1 °C min^-1^ to 210 °C (1 min hold), and finally 3 °C min^-1^ to 240 °C (2 min hold). The total analysis time per sample was approximately 34 min. An Agilent Chemstation was used for instrument control and data analysis. Data quantitation was carried out by matrix-matched calibration curve based on detected and integrated peak areas.

For target analyte confirmation using GC-MS/MS, an Agilent gas chromatograph (model 7890 N) connected to a 7890 N MSD, and equipped with an autosampler (7683) were utilized. The mass spectrometer was operated in positive ion mode, using SRM. Four transitions (2 quantifiers and 2 qualifiers) were monitored for native OCPs. The operating conditions were as follows: the injection port temperature (250 °C), injection volume (1 µL), injection mode (splitless), ion source temperature (230 °C), carrier gas (nitrogen, purity ≥ 99.999 %, at a flow rate = 1.2 mL min^-1^), respectively. The GC oven was operated with temperature conditions as follows: initial temperature: 80 °C (2 min), ramped at 20 °C min^-1^ to 200 °C min^-1^ (3 min), ramped at 5 °C min^-1^ to 250 °C min^-1^ (2 min), with a total run time of 23 min. The quadrupole mass spectromer was operated in dynamic reaction monitoring mode, scan from m/z 45 to 500. The emission current of the ionization filament was set at 15 µA with a dwell time of 55 ms. Target analytes were identified based on their retention times and qualitative ions (Supplementary Table 2). Data acquisition and processing were performed using Agilent Chemstation software.

**List of Supplementary Tables and Figures**

**S1 Table :** Chemical structures and properties of 20 organochlorine pesticide analytes studied.

**S2a Table :** GC-MS/MS instrumental conditions used in the analyses of ginseng

**S2b Table :** GC-µ-ECD instrumental conditions used in the analyses of ginseng

**S1 Figure**  Mean concentrations (n=3) quantified for endosulfans (α, β, and endosulfate sulfate) in ginseng using QuEChERS/GC-MS/MS and modified QuEChERS/sulphuric acid clean-up/GC-µ-ECD for low (0.1 mg kg^-1^) and high concentration (1 mg kg^-1^) uptake.

**S1 Table : Chemical structures and properties of 20 organochlorine pesticide analytes studied.**

| **Organochlorine**  **pesticide^a^** | **Molecular Weight (g/mol)^b^** | **Log Kow^c^** |
| --- | --- | --- |
|   Cis-Heptachlor epoxide | 389.3 | 5.1^e^ |
|   Trans-Heptachlor epoxide | 389.3 | 5.1^e^ |
|   o, p’- DDE | 318 | 5.43^d^ |
|   Alpha Endosulfan | 406.9 | 3.83^f^ |
|   p, p’- DDE | 318 | 5.7 |
|   Dieldrin | 380.9 | 5.20 |
|   o, p’- DDD | 320 | 4.87^d^ |

| **Organochlorine pesticide^a^** | **Molecular weight**  **(g mol^-1^)** | **Log Kow^c^** |
| --- | --- | --- |
|   Endrin | 380.9 | 5.20 |
|   β-Endosulfan | 406.9 | 3.62^f^ |
|   p, p’- DDD | 320 | 5.5 |
|   o, p’- DDT | 354.5 | 5.65^d^ |
|   Endosulfan sulphate | 422.9 | 3.66^f^ |
|   p, p’- DDT | 354.5 | 6.19 |

| **Organochlorine**  **pesticide^a^** | **Molecular Weight (g/mol)^b^** | **Log Kow^c^** |
| --- | --- | --- |
|  |  |  |
|   α-HCH | 290.8 | 3.81 |
|   HCB | 284.8 | 5.5 |
|   β-HCH | 290.8 | 3.80 |
|   γ-HCH | 290.8 | 3.70 |
|   δ-HCH | 290.8 | 4.14 |
|   Heptachlor | 373.3 | 5.27 |
|   Aldrin | 364.9 | 3.01 |

a: Organochlorine pesticide structures were constructed in ChemDraw version 19

b: OCP molecular weights were obtained from PubChem <https://pubchem.ncbi.nlm.nih.gov/>

c: [1]

d: [2]

e: [3]

f: [4]

**S2a Table : GC-MS/MS instrumental conditions used in the analyses of ginseng**

| Column | DB-5MS (30 m x 0.25 mm x 0.25 µm) | | | | | |
| --- | --- | --- | --- | --- | --- | --- |
| Carrier gas | N₂ (1.2 mL min^-1^) | | | | | |
| Injection vol. | 1 µL | | | | | |
| Injection Mode | Splitless | | | | | |
| Inlet temp. | 250 ℃ | | | | | |
| Oven temp. | Stage | Rate  (℃ min^-1^) | Temp (℃) | Hold time  (min) | | Run time  (min) |
|  | Initial | - | 80 | 2 | | 1 |
|  | Ramp 1 | 20 | 200 | 3 | | 11 |
|  | Ramp 2 | 5 | 250 | 2 | | 23 |
| Ion source | EI | | Ion source temp. | | 230 ℃ | |
| Electron energy(eV) | 70 | | Scan type | DMRM | | |

**S2b Table : GC-µ-ECD instrumental conditions used in the analyses of ginseng**

| Column | | DB-5MS (30 m x 0.25 mm x 0.25 µm) | | | | |  |
| --- | --- | --- | --- | --- | --- | --- | --- |
| Carrier gas | | N₂ (6 mL min^-1^) | | | | |  |
| Injection vol. | | 1 µL | | | | |  |
| Injection Mode | | Splitless | | | | |  |
| Inlet temp. | | 250 ℃ | | | | |  |
|  | Stage | | Rate  (°C min^-1^) | Temp (°C) | Hold time (min) | Run time (min) | |
|  | Initial | |  | 80 | 2 |  | |
|  | Ramp 1 | | 20 | 180 | 2 |  | |
|  | Ramp 2 | | 5 | 205 | 2 |  | |
| Oven temp | Ramp 3 | | 1 | 207 | 3 |  | |
|  | Ramp 4 | | 1 | 210 | 3 |  | |
|  | Ramp 5 | | 3 | 240 | 10 | 34 | |

Concentration (mg kg^-1^)

**S1 Figure** Mean concentrations (n=3) quantified for endosulfans (α, β, and endosulfate sulfate) in ginseng using QuEChERS/GC-MS/MS and modified QuEChERS/sulphuric acid clean-up/GC-µ-ECD for low (0.1 mg kg^-1^) and high concentration (1 mg kg^-1^) uptake.

References

1. Mackay, D., W.-Y. Shiu, and S.C. Lee, *Handbook of physical-chemical properties and environmental fate for organic chemicals*. 2006, CRC press.

2. Finizio, A., M. Vighi, and D. Sandroni, *Determination of n-octanol/water partition coefficient (Kow) of pesticide critical review and comparison of methods.* Chemosphere, 1997. **34**: p. 131-161.

3. Meador, J., et al., *Comparative bioaccumulation of chlorinated hydrocarbons from sediment by two infaunal invertebrates.* Arch Environ Con Tox, 1997. **33**(4): p. 388-400.

4. Agency for Toxic Substances and Disease Registry, *Endosulfan: Toxicological Profile for Endosulfan*. 1997.
